# Supplementary material for: Estimating intraspecific genetic diversity from community DNA metabarcoding data
Source: PeerJ. 2018 Apr 9;6:e4644. doi: 10.7717/peerj.4644 (PMC5896493; doi:10.7717/peerj.4644)
Supplement: Supplemental Information 1 — Overview of the metabarcoding process, with key biases potentially affecting sequence accuracy (shown in red). In the bulk sample (A) several species with different biomass (indicated by circle size) and distinct haplotypes (indicated by colour) are present. After tissue homogenization and DNA extraction the COI marker is amplified using PCR (B), which can not only skew sequence abundance but also fail to amplify taxa due to primer bias (Elbrecht & Leese, 2015) or insufficient sequencing depth in the case of underrepresented/rare taxa (Elbrecht, Peinert & Leese, 2017). In the process of HTS (C) many new false sequence variants are generated due to sequencing errors (Schirmer et al., 2015), chimera formation (Edgar et al., 2011) and mixing of multiplexed samples (Esling, Lejzerowicz & Pawlowski, 2015; Schnell, Bohmann & Gilbert, 2015). The impact of these errors is usually reduced by strict quality filtering and clustering of similar sequences into operational taxonomic units (OTUs). Normally, only the most abundant sequence in an OTU is considered and used to identify the respective species, which in turn means that information on genetic diversity is lost (Callahan, McMurdie & Holmes, 2017) (D). Recently alternative denoising strategies have been developed to remove sequences affected by errors from a dataset and retain the actual haplotype sequences present in a sample (Eren et al., 2015; Edgar & Flyvbjerg, 2015; Callahan et al., 2016; Amir et al., 2017). Figure based on Figure S1 in Callahan et al., 2016. [file peerj-06-4644-s001.pdf]

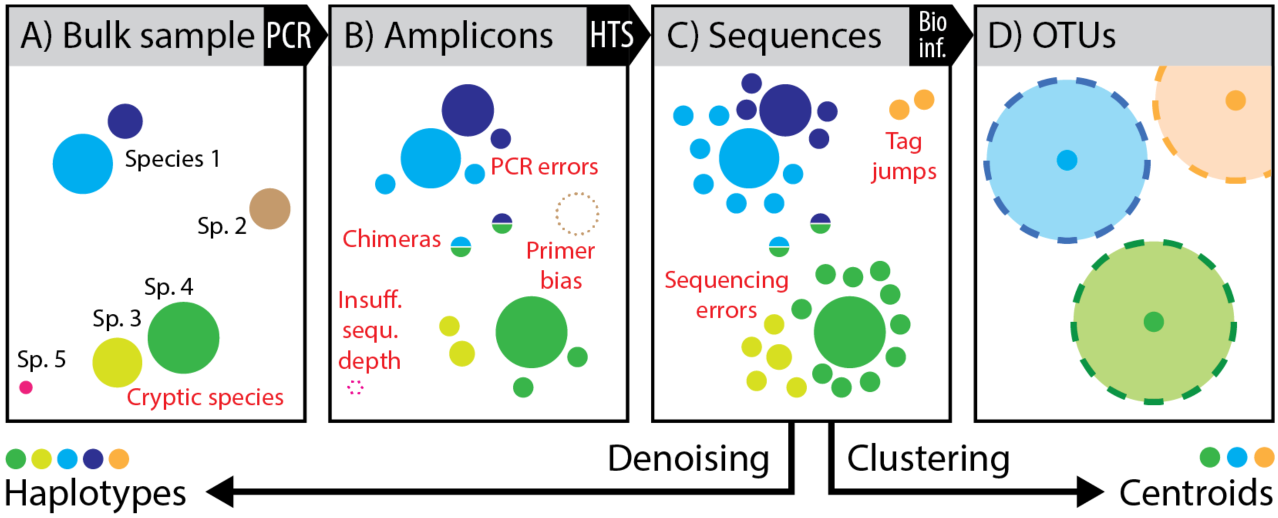

Figure S1: Overview of the metabarcoding process, with key biases potentially affecting sequence accuracy (shown in red). In the bulk sample (**A**) several species with different biomass (indicated by circle size) and distinct haplotypes (indicated by colour) are present. After tissue homogenization and DNA extraction the COI marker is amplified using PCR (**B**), which can not only skew sequence abundance but also fail to amplify taxa due to primer bias (Elbrecht & Leese, 2015) or insufficient sequencing depth in the case of underrepresented / rare taxa (Elbrecht, Peinert & Leese, 2017). In the process of HTS (**C**) many new false sequence variants are generated due to sequencing errors (Schirmer et al., 2015), chimera formation (Edgar et al., 2011) and mixing of multiplexed samples (Esling, Lejzerowicz & Pawlowski, 2015; Schnell, Bohmann & Gilbert, 2015). The impact of these errors is usually reduced by strict quality filtering and clustering of similar sequences into operational taxonomic units (OTUs). Normally, only the most abundant sequence in an OTU is considered and used to identify the respective species, which in turn means that information on genetic diversity is lost (Callahan, McMurdie & Holmes, 2017) (**D**). Recently alternative denoising strategies have been developed to remove sequences affected by errors from a dataset and retain the actual haplotype sequences present in a sample (Eren et al., 2015; Edgar & Flyvbjerg, 2015; Callahan et al., 2016; Amir et al., 2017). Figure based on Figure S1 in Callahan *et al*. 2016.

References:

Amir A, McDonald D, Navas-Molina JA, Kopylova E, Morton JT, Zech Xu Z, Kightley EP, Thompson LR, Hyde ER, Gonzalez A, Knight R 2017. Deblur Rapidly Resolves Single-Nucleotide Community Sequence Patterns. *mSystems* 2:e00191–16–7. DOI: 10.1128/mSystems.00191-16.

Callahan BJ, McMurdie PJ, Holmes SP 2017. Exact sequence variants should replace operational taxonomic units in marker-gene data analysis. :1–5. DOI: 10.1038/ismej.2017.119.

Callahan BJ, McMurdie PJ, Rosen MJ, Han AW, Johnson AJA, Holmes SP 2016. DADA2: High-resolution sample inference from Illumina amplicon data. *Nature Methods* 13:581–583. DOI: 10.1038/nmeth.3869.

Edgar RC, Flyvbjerg H 2015. Error filtering, pair assembly and error correction for next-generation sequencing reads. *Bioinformatics* 31:3476–3482. DOI: 10.1093/bioinformatics/btv401.

Edgar RC, Haas BJ, Clemente JC, Quince C, Knight R 2011. UCHIME improves sensitivity and speed of chimera detection. *Bioinformatics* 27:2194–2200. DOI: 10.1093/bioinformatics/btr381.

Elbrecht V, Leese F 2015. Can DNA-Based Ecosystem Assessments Quantify Species Abundance? Testing Primer Bias and Biomass—Sequence Relationships with an Innovative Metabarcoding Protocol. *PloS one* 10:e0130324–16. DOI: 10.1371/journal.pone.0130324.

Elbrecht V, Peinert B, Leese F 2017. Sorting things out: Assessing effects of unequal specimen biomass on DNA metabarcoding. *Ecology and Evolution* 7:6918–6926. DOI: 10.1002/ece3.3192.

Eren AM, Morrison HG, Lescault PJ, Reveillaud J, Vineis JH, Sogin ML 2015. Minimum entropy decomposition: Unsupervised oligotyping for sensitive partitioning of high- throughput marker gene sequences. 9:968–979. DOI: 10.1038/ismej.2014.195.

Esling P, Lejzerowicz F, Pawlowski J 2015. Accurate multiplexing and filtering for high-throughput amplicon-sequencing. *Nucleic acids research* 43:2513–2524. DOI: 10.1093/nar/gkv107.

Schirmer M, Ijaz UZ, D'Amore R, Hall N, Sloan WT, Quince C 2015. Insight into biases and sequencing errors for amplicon sequencing with the Illumina MiSeq platform. *Nucleic acids research*:1–16. DOI: 10.1093/nar/gku1341.

Schnell IB, Bohmann K, Gilbert MTP 2015. Tag jumps illuminated - reducing sequence-to-sample misidentifications in metabarcoding studies. *Molecular ecology resources* 15:1289–1303. DOI: 10.1111/1755-0998.12402.
