# Supplementary material for: Estimating intraspecific genetic diversity from community DNA metabarcoding data
Source: PeerJ. 2018 Apr 9;6:e4644. doi: 10.7717/peerj.4644 (PMC5896493; doi:10.7717/peerj.4644)
Supplement: Supplemental Information 2 — Detailed bioinformatic processing of metabarcoding to extract haplotype sequences using the JAMP R package. A) Metabarcoding raw data is processed and quality filtered. These steps are integrated in JAMP, but most other standard metabarcoding pipelines could be used as well. B) The processed and quality filtered samples from step A would be usually clustered into operational taxonomic units, but are here additionally filtered (retaining reads of only the expected amplicon length and discarding reads of low abundance) and then denoised. C) In denoising with usearch unoise3 the strictness of denoising is controlled by the alpha value (low alpha = less noise, however more true haplotypes get discarded). D) The denoised reads (=haplotypes) are clustered into OTUs grouped by similarity and the abundance of each haplotype for each sample is exported in a table. E) The haplotype table is additionally filtered using different thresholds, to reduce the presence of low abundant OTUs and haplotypes and increase data reliability. F) The final filtered haplotype table can be used for phylogeographic and population genetic analysis. [file peerj-06-4644-s002.pdf]

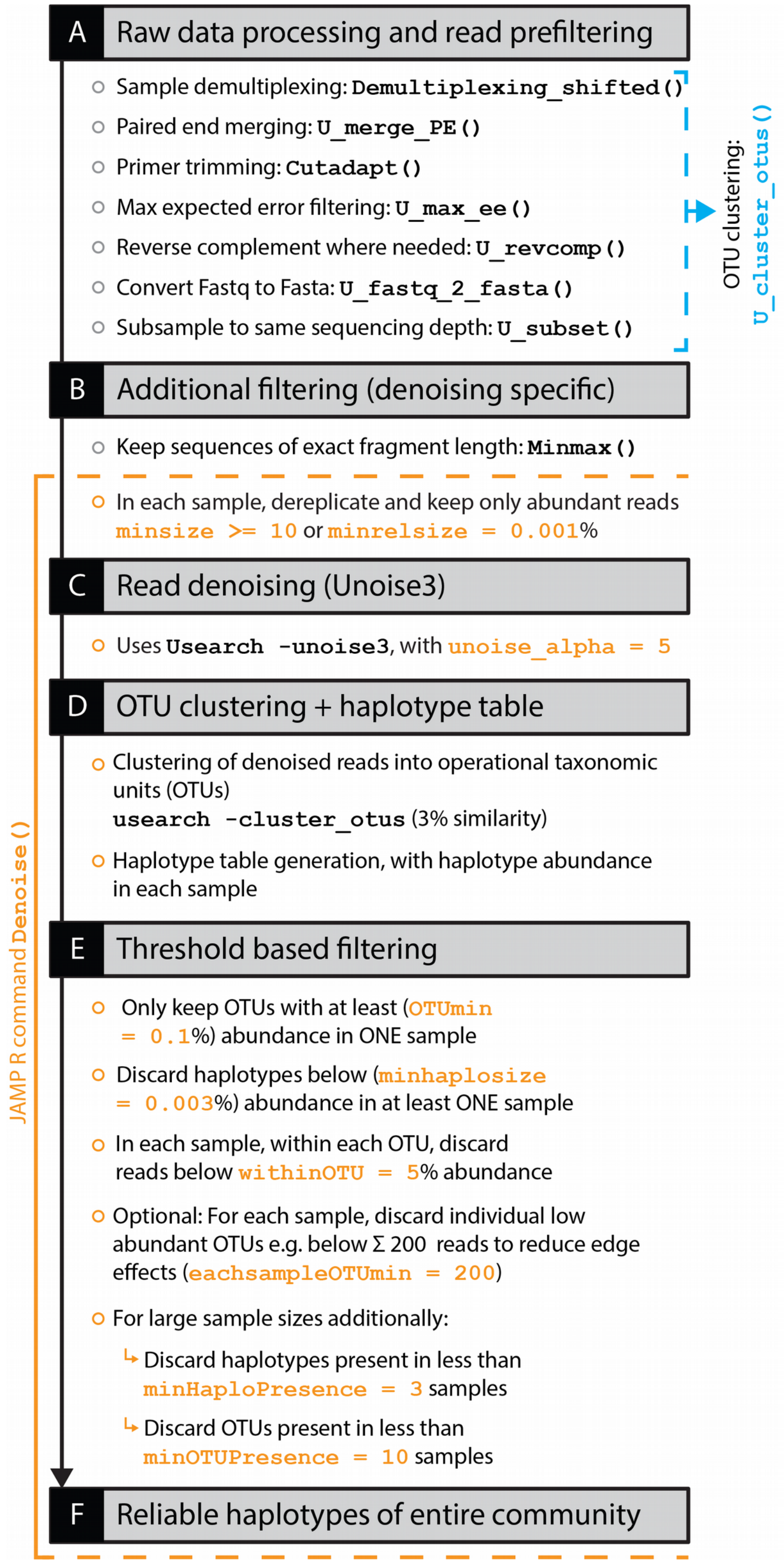

Figure S2: Detailed bioinformatic processing of metabarcoding to extract haplotype sequences using the JAMP R package. **A**) Metabarcoding raw data is processed and quality filtered. These steps are integrated in JAMP, but most other standard metabarcoding pipelines could be used as well. **B**) The processed and quality filtered samples from step A would be usually clustered into operational taxonomic units, but are here additionally filtered (retaining reads of only the expected amplicon length and discarding reads of low abundance) and then denoised. **C**) In denoising with usearch unoise3 the strictness of denoising is controlled by the alpha value (low alpha = less noise, however more true haplotypes get discarded). **D**) The denoised reads (=haplotypes) are clustered into OTUs grouped by similarity and the abundance of each haplotype for each sample is exported in a table. **E**) The haplotype table is additionally filtered using different thresholds, to reduce the presence of low abundant OTUs and haplotypes and increase data reliability. **F**) The final filtered haplotype table can be used for phylogeographic and population genetic analysis.
