# Supplementary material for: Estimating intraspecific genetic diversity from community DNA metabarcoding data
Source: PeerJ. 2018 Apr 9;6:e4644. doi: 10.7717/peerj.4644 (PMC5896493; doi:10.7717/peerj.4644)
Supplement: Supplemental Information 4 — Effect of different haplotype recovery of in the single species mock sample, when using different alpha values with Unoise3 (as integrated in the JAMP package). Not all reads are shared between both replicates (indicated by A or B instead of a circle). The 15 expected haplotypes are shown in black, while unexpected ones are highlighted in gray or blue. Error bars show the standard deviation of relative read abundance between both replicates, for the respective haplotype. [file peerj-06-4644-s004.pdf]

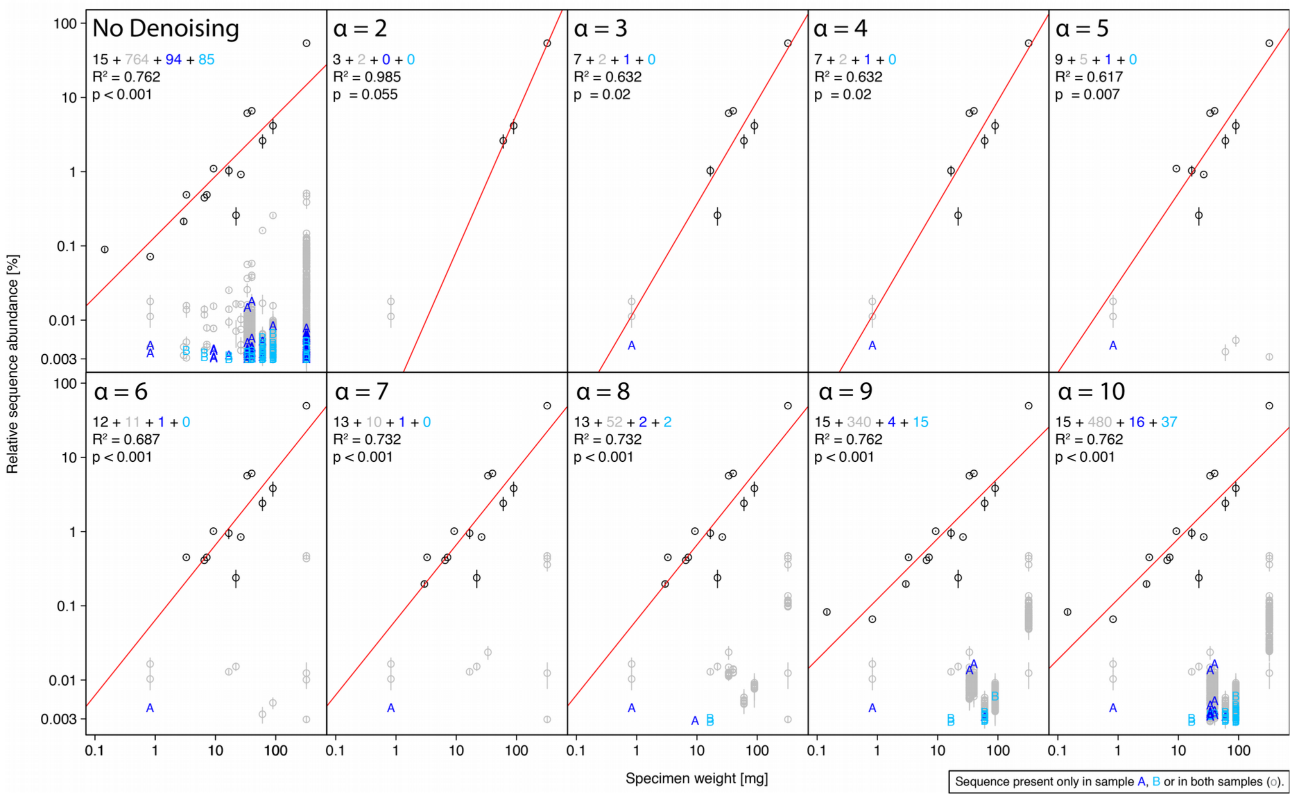

Figure S4: Effect of different haplotype recovery of in the single species mock sample, when using different alpha values with Unoise3 (as integrated in the JAMP package). Not all reads are shared between both replicates (indicated by A or B instead of a circle). The 15 expected haplotypes are shown in black, while unexpected ones are highlighted in gray or blue. Error bars show the standard deviation of relative read abundance between both replicates, for the respective haplotype.
