# Supplementary material for: Estimating intraspecific genetic diversity from community DNA metabarcoding data
Source: PeerJ. 2018 Apr 9;6:e4644. doi: 10.7717/peerj.4644 (PMC5896493; doi:10.7717/peerj.4644)
Supplement: Supplemental Information 5 — Bar plots showing the haplotype composition of all 199 OTUs obtained with the BF2+BR2 primer combination. The OTU number is indicated above each bar, with the four taxa shown in Figure 2 being highlighted. Haplotypes are shown in different colours, with white bars indicating the proportion of sites where the respective OTU was not detected. Most OTUs were only present at a few sample sites. [file peerj-06-4644-s005.pdf]

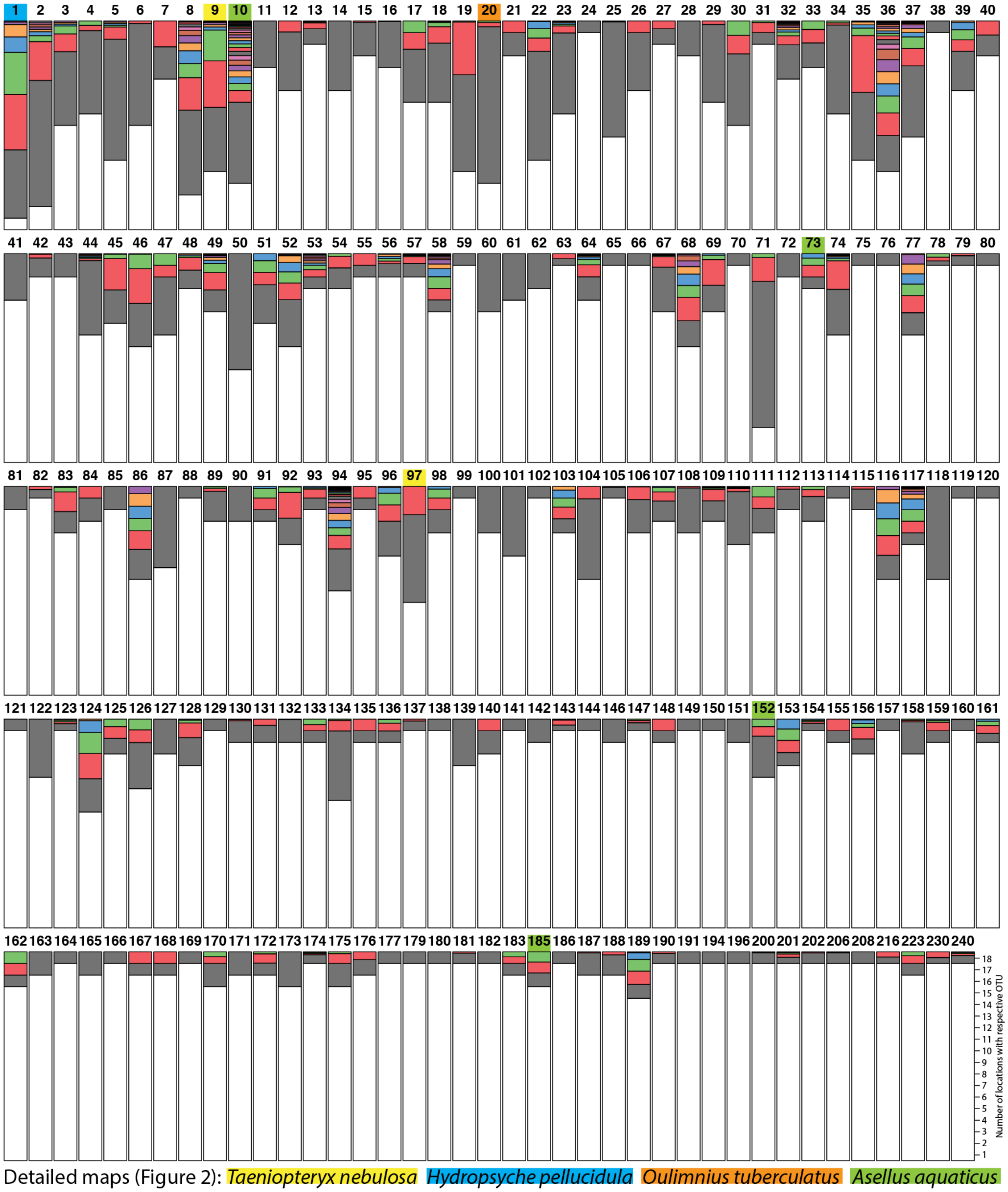

Figure S5: Bar plots showing the haplotype composition of all 199 OTUs obtained with the BF2+BR2 primer combination. The OTU number is indicated above each bar, with the four taxa shown in Figure 2 being highlighted. Haplotypes are shown in different colours, with white bars indicating the proportion of sites where the respective OTU was not detected. Most OTUs were only present at a few sample sites.
