# Supplementary material for: Estimating intraspecific genetic diversity from community DNA metabarcoding data
Source: PeerJ. 2018 Apr 9;6:e4644. doi: 10.7717/peerj.4644 (PMC5896493; doi:10.7717/peerj.4644)

Figure S6: Detailed haplotype maps, networks and sequence alignment for all 4 primer combinations and replicates of selected taxa. **a)** Haplotype maps for both replicates for each of the four primer combinations. For *A. aquaticus* only the 10 most common haplotypes are shown in different colours (remaining ones in white). For each primer combination, the haplotypes in the map and network have the same corresponding colours. **b)** Haplotype networks for each primer pair. Each cross line represents one base pair difference between the respective haplotypes. Haplotypes present in just one replicate are indicated by A or B next to the network node. Dashed lines around a circle indicate novel haplotypes that were not available in the BOLD reference database. **c)** Quantification of similarity between both replicates, by plotting abundance of individual haplotypes of each sampling point against each other. The red line indicates the best fit (with significance and adjusted R^2 value given in each plot). **d)** Sequence alignment of all haplotypes, with mismatching nucleotides between sequences highlighted (green = T, red = A, yellow = G and blue = C). Shapefile-data © OpenStreetMap contributors, licensed under Creative Commons 2.0 (CC BY-SA).

See the following pages for example plots of:
Page 2: *Taeniopteryx nebulosa*
Page 3: *Hydropsyche pellucidula*
Page 4: *Oulimnius tuberculatus*
Page 5: *Asellus aquaticus*

I) ***Taeniopteryx nebulosa*** (Plecoptera)

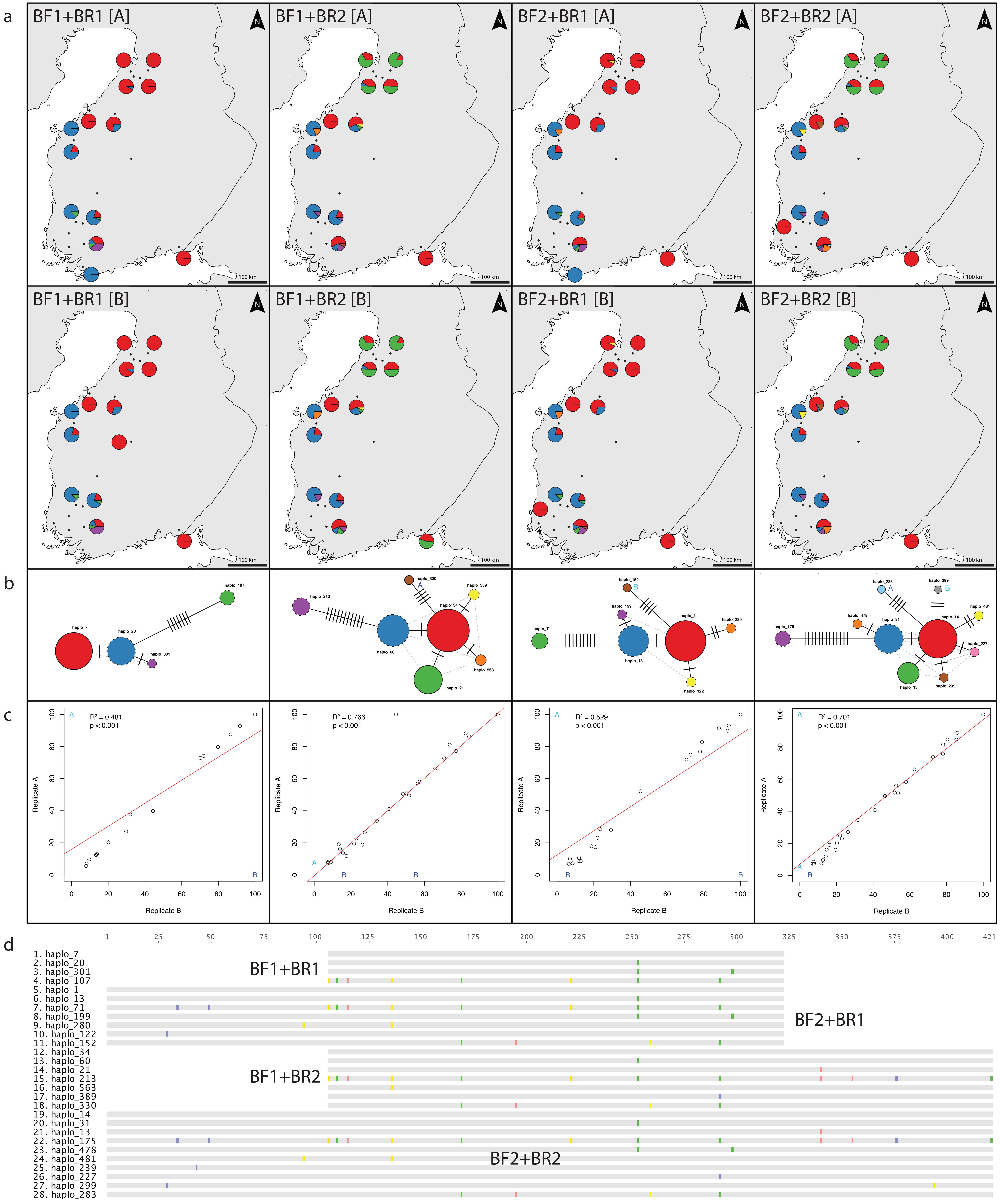

II) *Hydropsyche pellucidula* (Trichoptera)

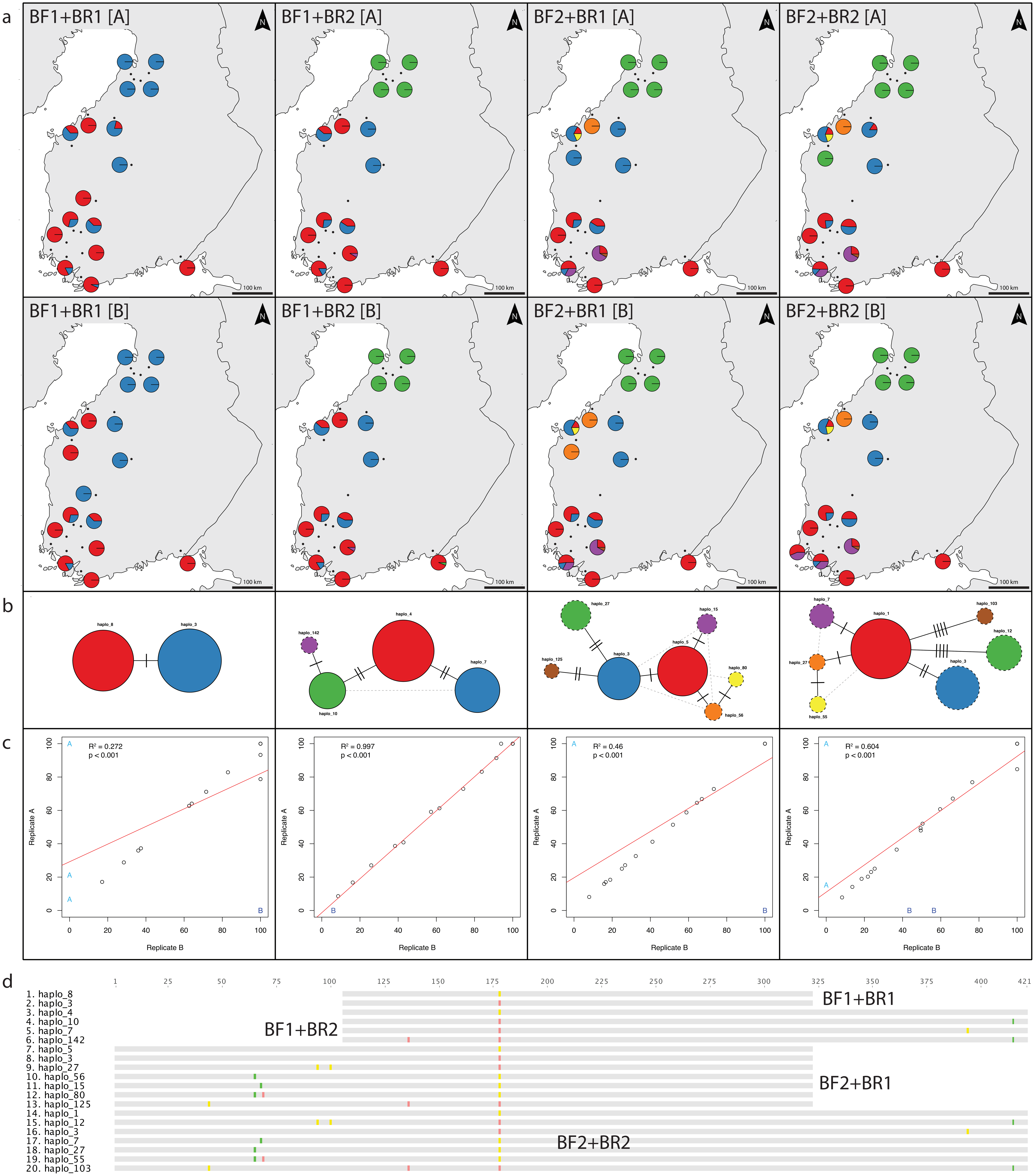

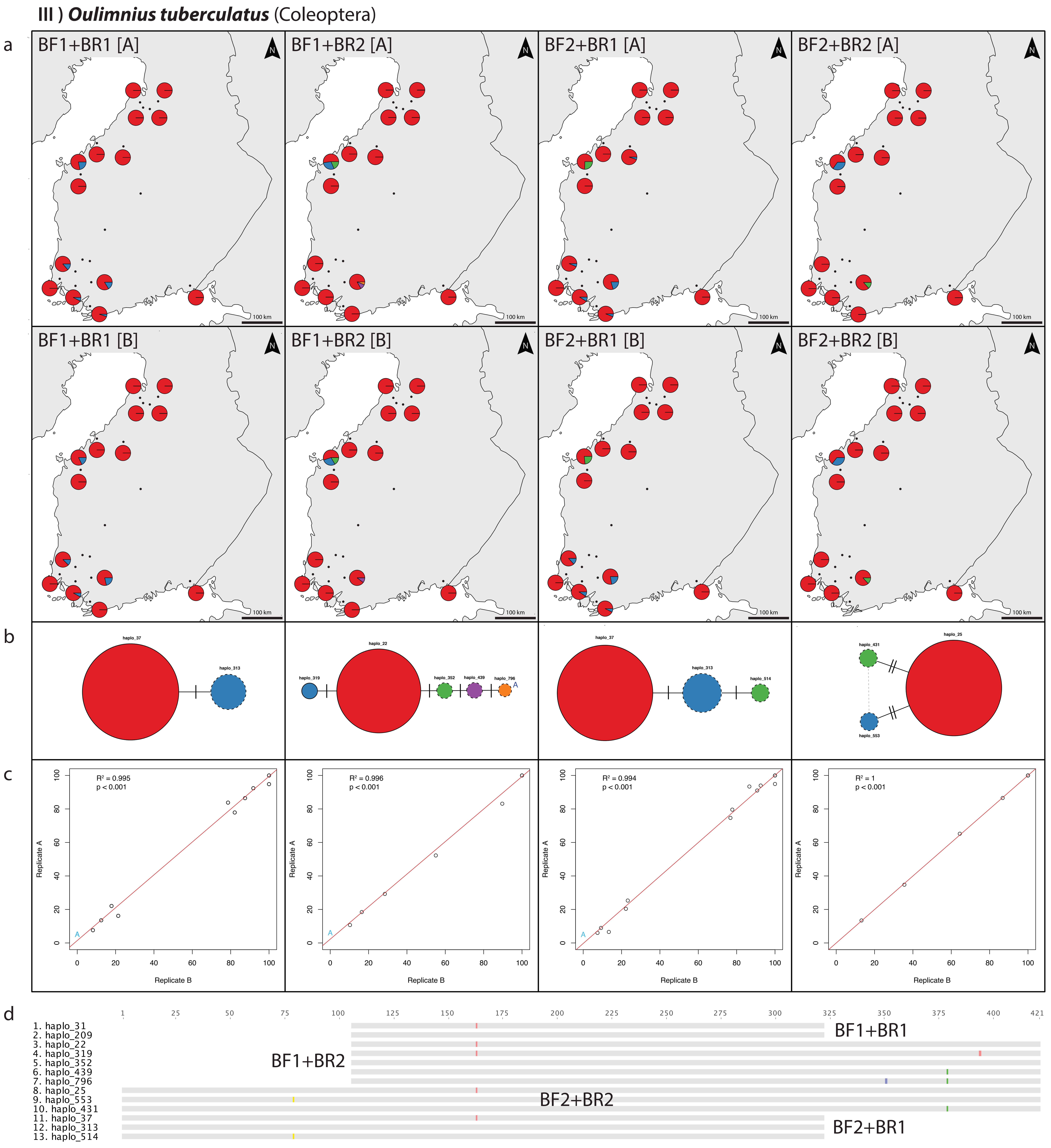
III) Oulimnius tuberculatus (Coleoptera)
a
BF1+BR1 [A]
BF1+BR2 [A]
BF2+BR1 [A]
BF2+BR2 [A]
BF1+BR1 [B]
BF1+BR2 [B]
BF2+BR1 [B]
BF2+BR2 [B]
100 km
b
haplo_37
haplo_313
haplo_319
haplo_22
haplo_352
haplo_439
haplo_796
haplo_37
haplo_313
haplo_514
haplo_431
haplo_25
haplo_553
c
R² = 0.995
p < 0.001
R² = 0.996
p < 0.001
R² = 0.994
p < 0.001
R² = 1
p < 0.001
Replicate A
Replicate B
d
1. haplo_31
2. haplo_209
3. haplo_22
4. haplo_319
5. haplo_352
6. haplo_439
7. haplo_796
8. haplo_25
9. haplo_553
10. haplo_431
11. haplo_37
12. haplo_313
13. haplo_514
BF1+BR1
BF1+BR2
BF2+BR2
BF2+BR1

IV) *Asellus aquaticus* (Isopoda)

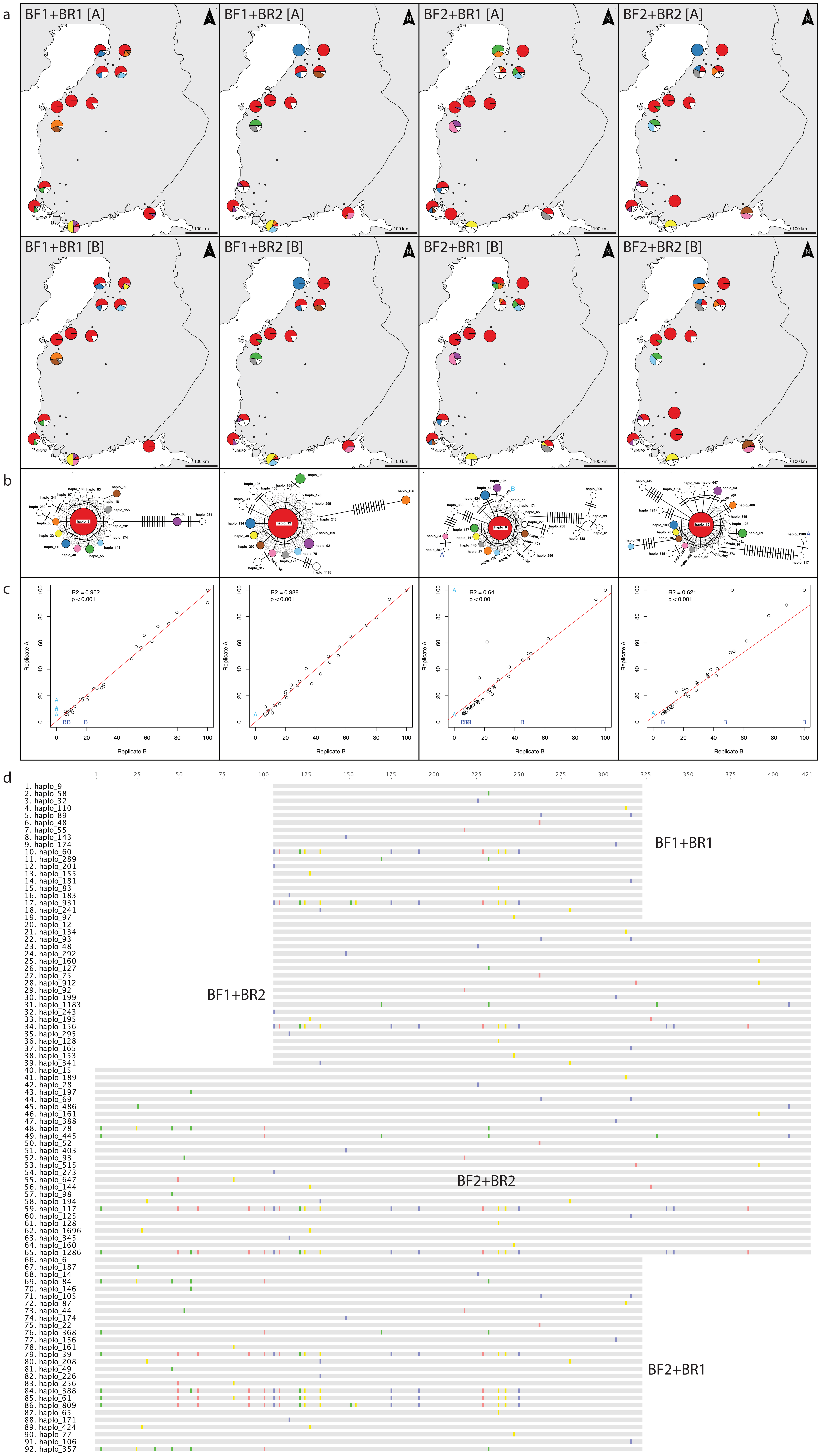

Supplement: Supplemental Information 6 — Detailed haplotype maps, networks and sequence alignment for all 4 primer combinations and replicates of selected taxa. a) Haplotype maps for both replicates for each of the four primer combinations. For A. aquaticus only the 10 most common haplotypes are shown in different colours (remaining ones in white). For each primer combination, the haplotypes in the map and network have the same corresponding colours. b) Haplotype networks for each primer pair. Each cross line represents one base pair difference between the respective haplotypes. Haplotypes present in just one replicate are indicated by A or B next to the network node. Dashed lines around a circle indicate novel haplotypes that were not available in the BOLD reference database. c) Quantification of similarity between both replicates, by plotting abundance of individual haplotypes of each sampling point against each other. The red line indicates the best fit (with significance and adjusted R2 value given in each plot). d) Sequence alignment of all haplotypes, with mismatching nucleotides between sequences highlighted (green = T, red = A, yellow = G and blue = C). See the following pages for example plots of: Page 2: Taeniopteryx nebulosa Page 3: Hydropsyche pellucidula Page 4: Oulimnius tuberculatus Page 5: Asellus aquaticus. [file peerj-06-4644-s006.pdf]
