# Supplementary figures and images for: Estimating intraspecific genetic diversity from community DNA metabarcoding data
Source: PeerJ. 2018 Apr 9;6:e4644. doi: 10.7717/peerj.4644 (PMC5896493; doi:10.7717/peerj.4644)

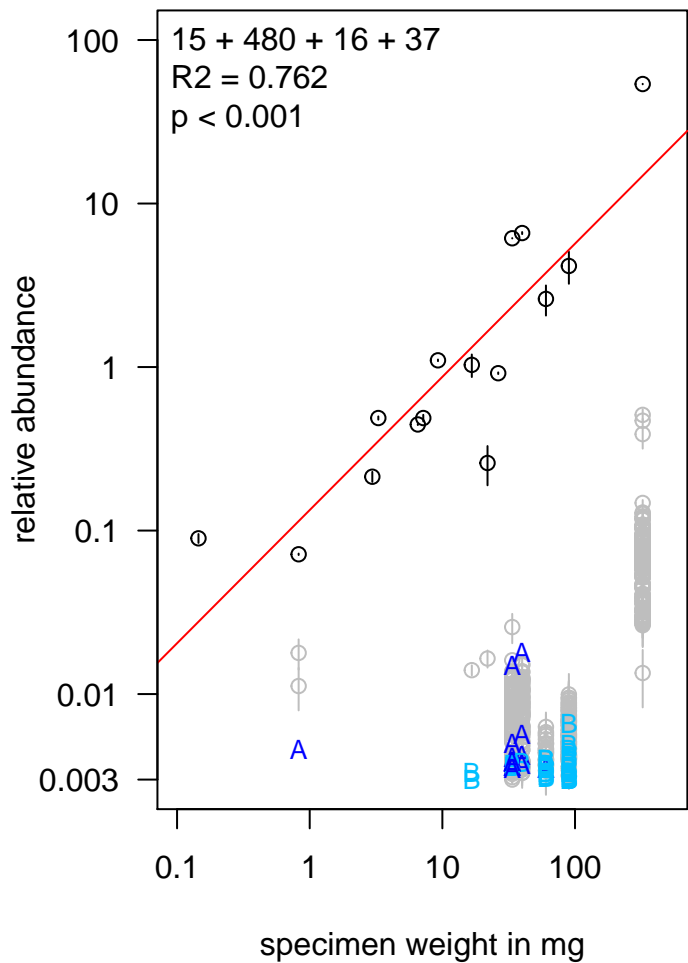

Supplement: Supplemental Information 8 [file peerj-06-4644-s008.zip › Scripts_S1_v3/1_Single_species_mock/R_alpha_values!/3_tables/DceM_alpha_10.pdf]

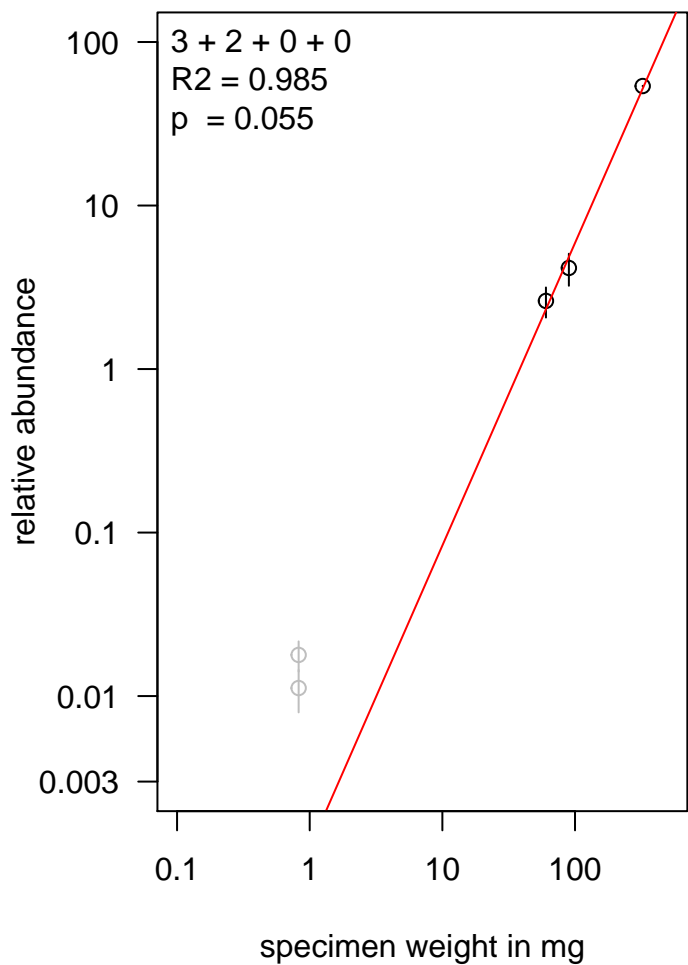

Supplement: Supplemental Information 8 [file peerj-06-4644-s008.zip › Scripts_S1_v3/1_Single_species_mock/R_alpha_values!/3_tables/DceM_alpha_2.pdf]

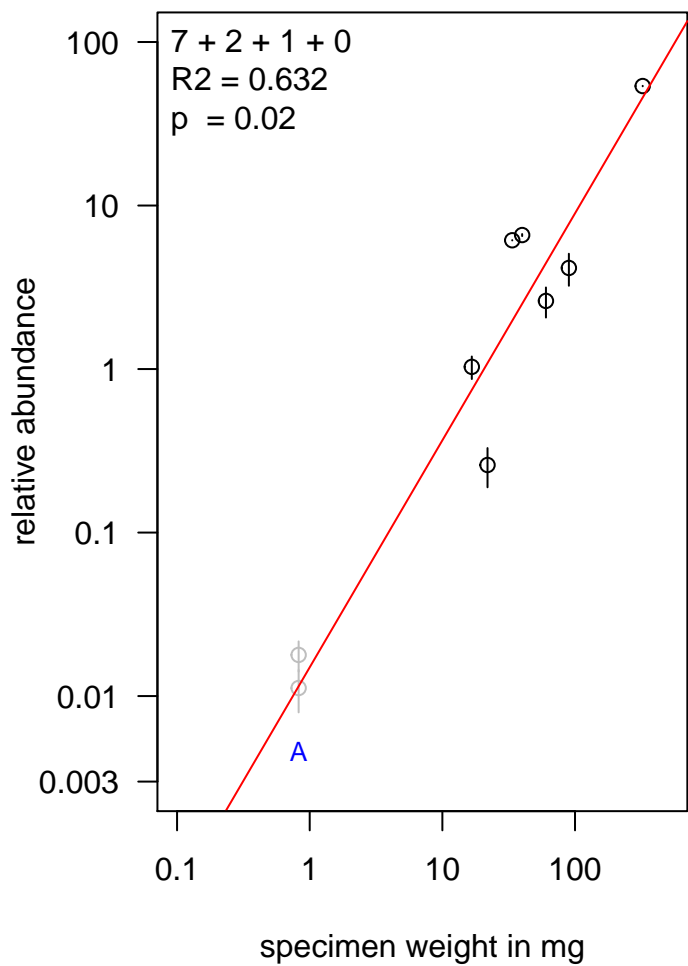

Supplement: Supplemental Information 8 [file peerj-06-4644-s008.zip › Scripts_S1_v3/1_Single_species_mock/R_alpha_values!/3_tables/DceM_alpha_3.pdf]

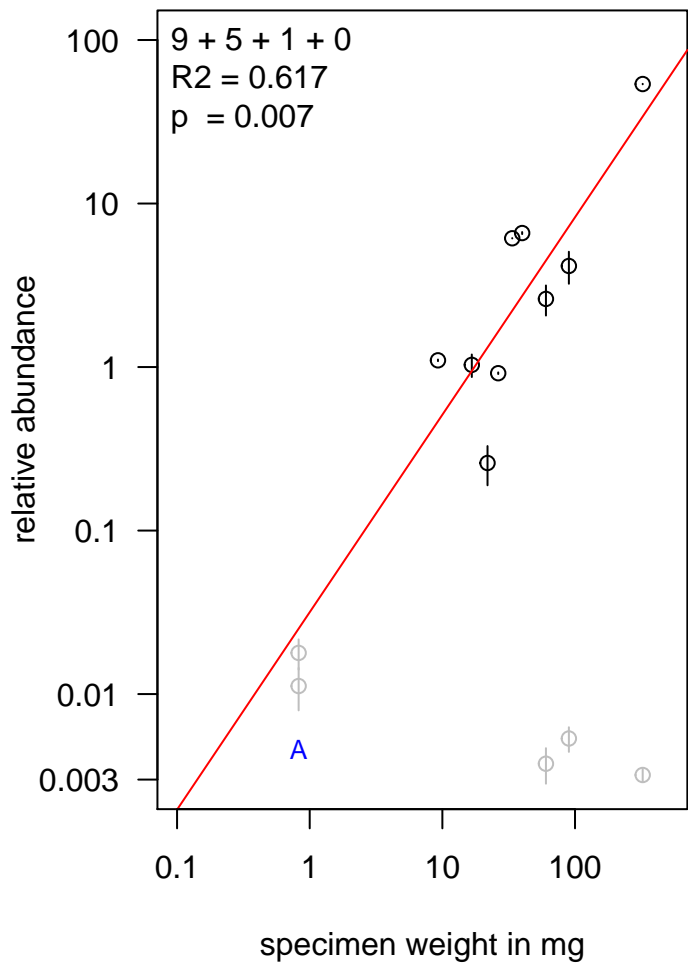

Supplement: Supplemental Information 8 [file peerj-06-4644-s008.zip › Scripts_S1_v3/1_Single_species_mock/R_alpha_values!/3_tables/DceM_alpha_5.pdf]

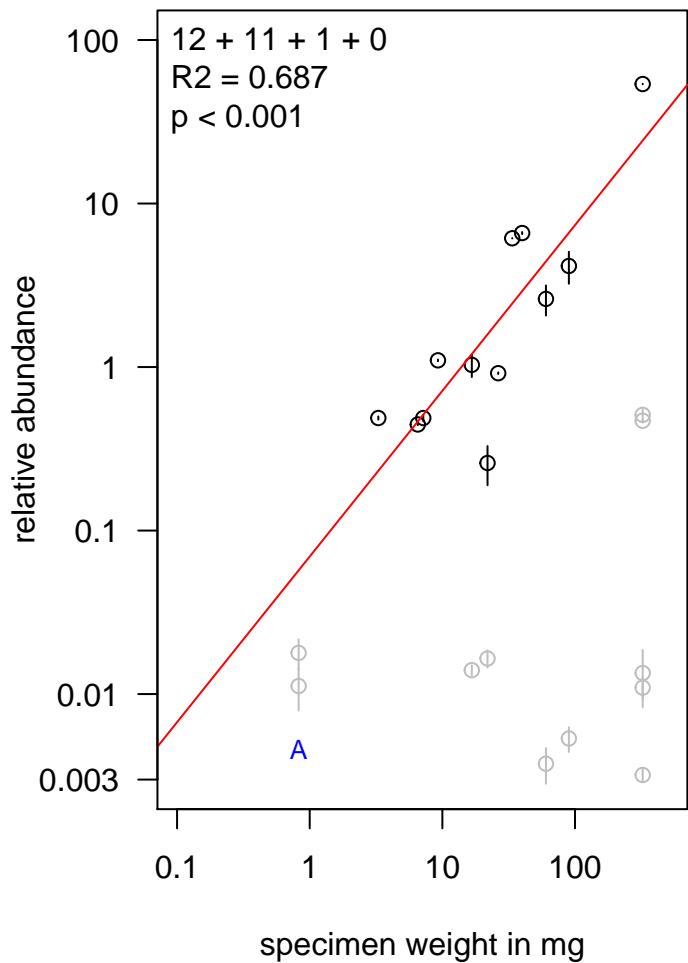

Supplement: Supplemental Information 8 [file peerj-06-4644-s008.zip › Scripts_S1_v3/1_Single_species_mock/R_alpha_values!/3_tables/DceM_alpha_6.pdf]

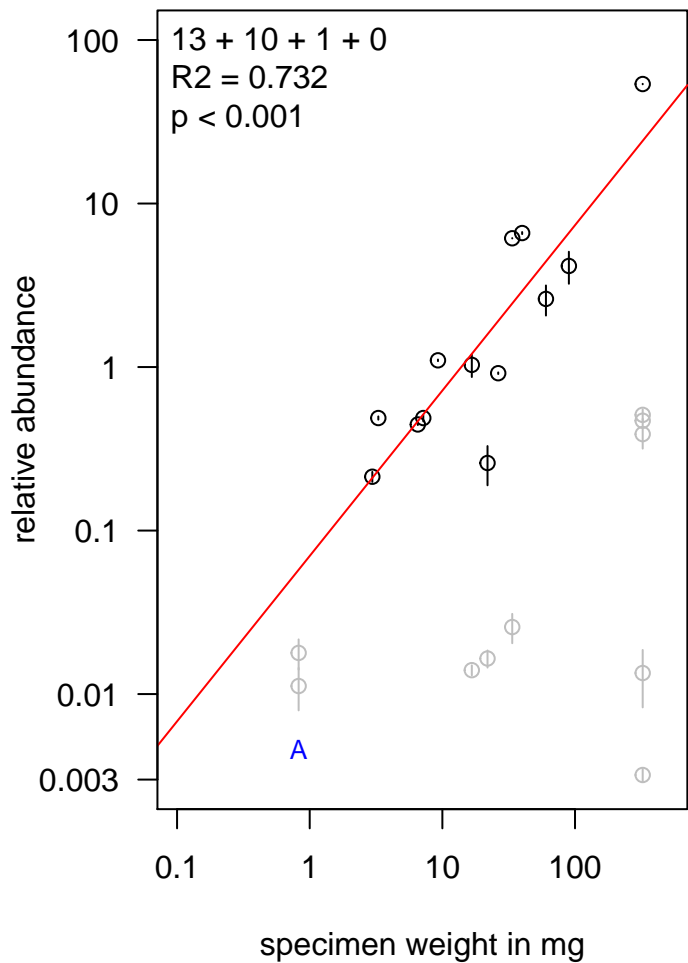

Supplement: Supplemental Information 8 [file peerj-06-4644-s008.zip › Scripts_S1_v3/1_Single_species_mock/R_alpha_values!/3_tables/DceM_alpha_7.pdf]

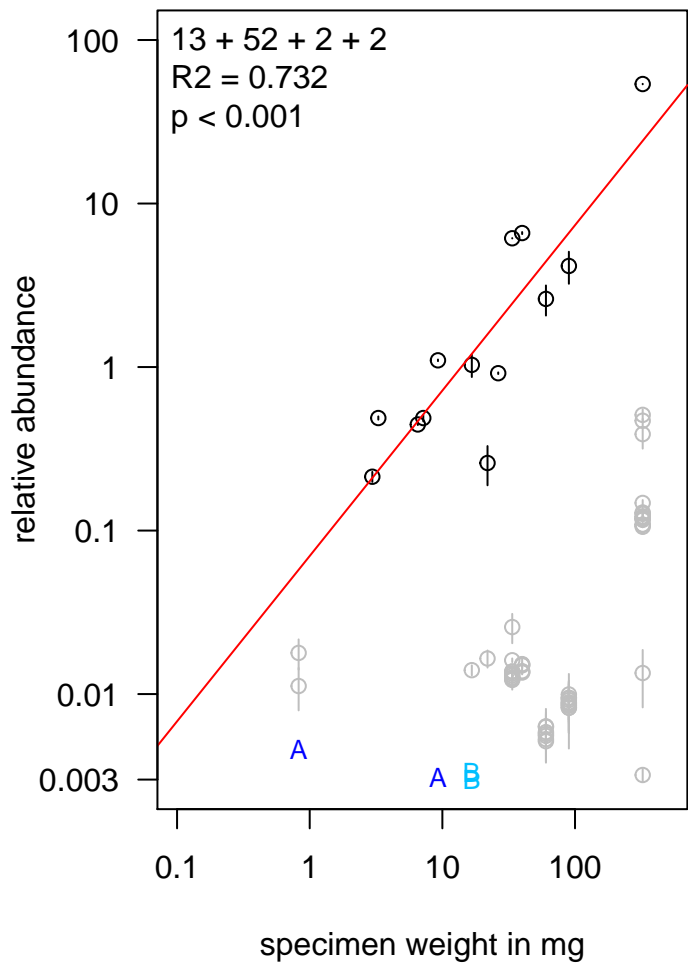

Supplement: Supplemental Information 8 [file peerj-06-4644-s008.zip › Scripts_S1_v3/1_Single_species_mock/R_alpha_values!/3_tables/DceM_alpha_8.pdf]

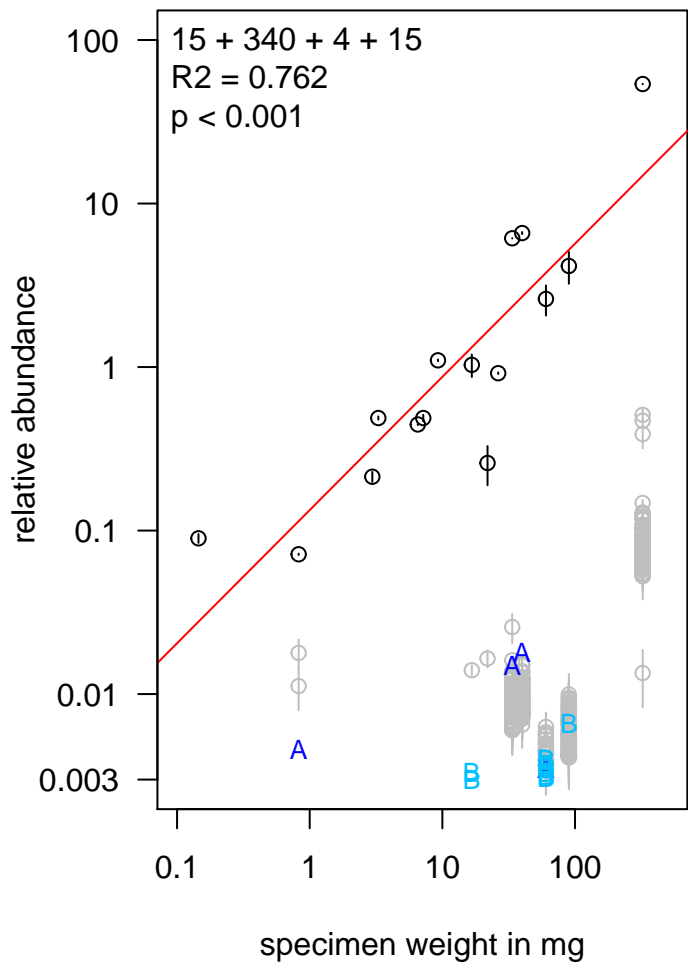

Supplement: Supplemental Information 8 [file peerj-06-4644-s008.zip › Scripts_S1_v3/1_Single_species_mock/R_alpha_values!/3_tables/DceM_alpha_9.pdf]

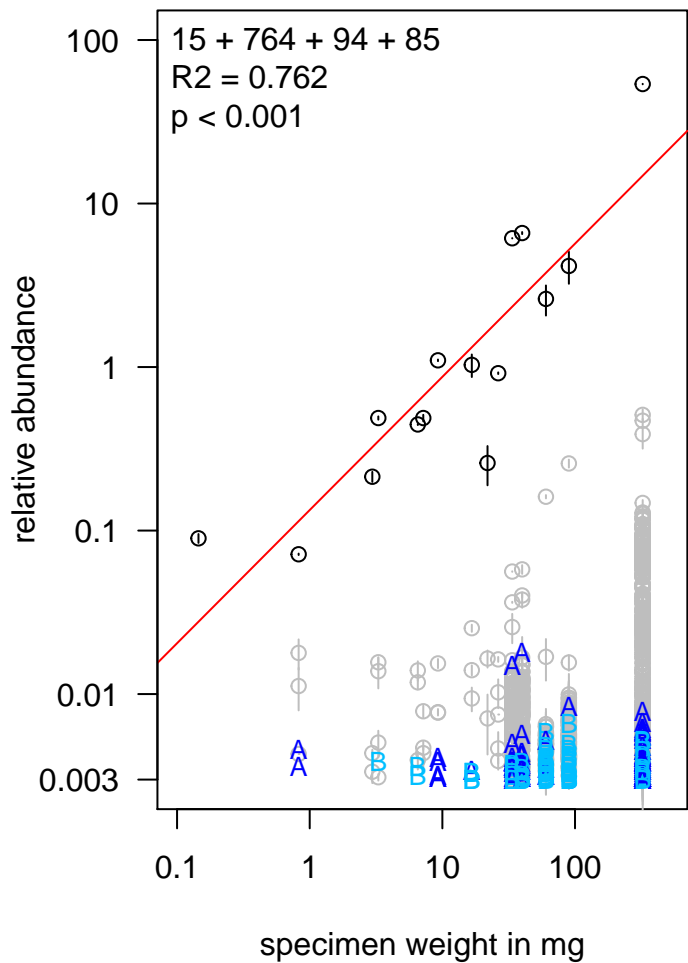

Supplement: Supplemental Information 8 [file peerj-06-4644-s008.zip › Scripts_S1_v3/1_Single_species_mock/R_alpha_values!/4_DceM__ee0.5_noDenoise.pdf]

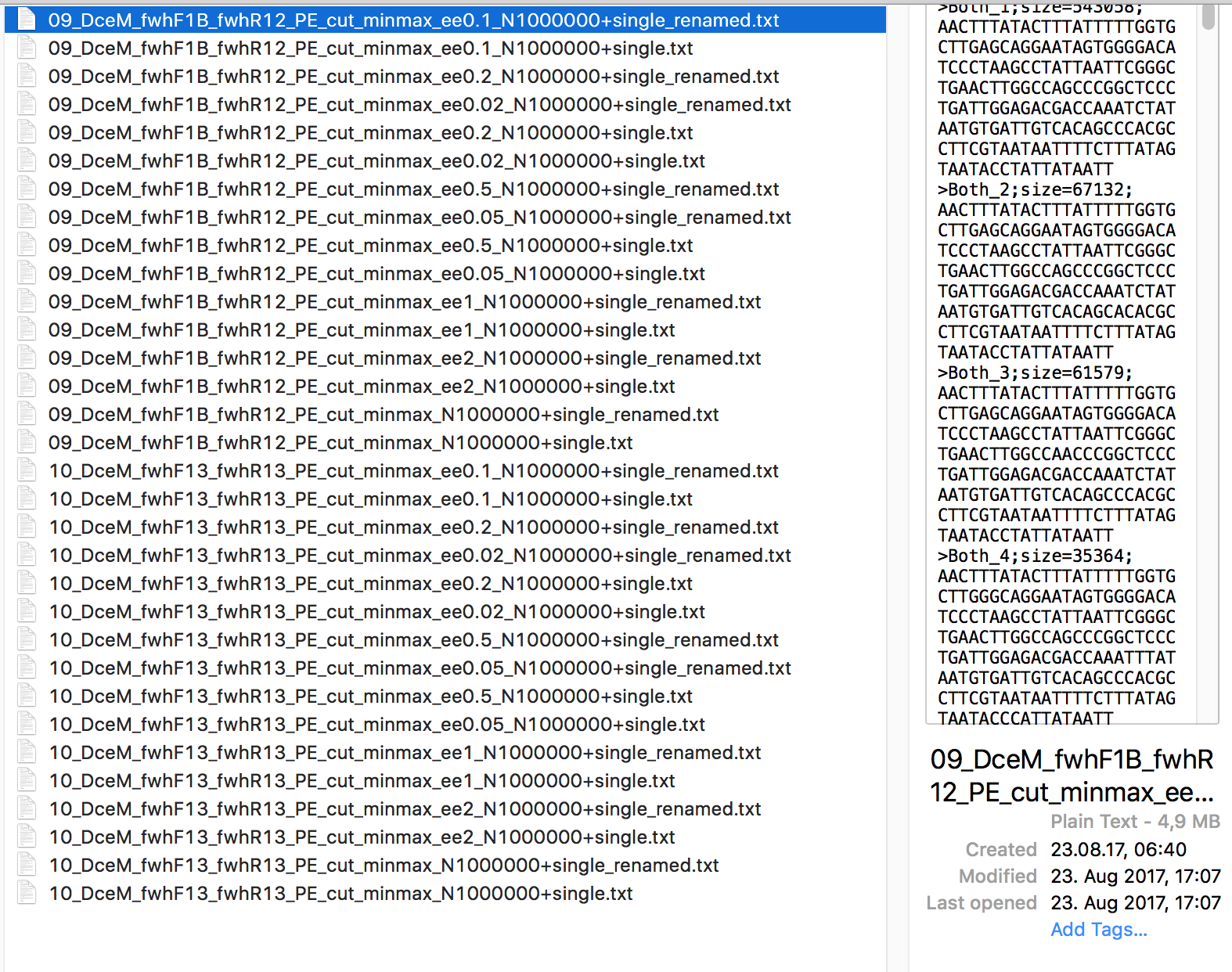

Supplement: Supplemental Information 8 [file peerj-06-4644-s008.zip › Scripts_S1_v3/1_Single_species_mock/R_dif_ee_filtering/1_imputfiles/Screen Shot 2018-03-14 at 09.21.32.png]

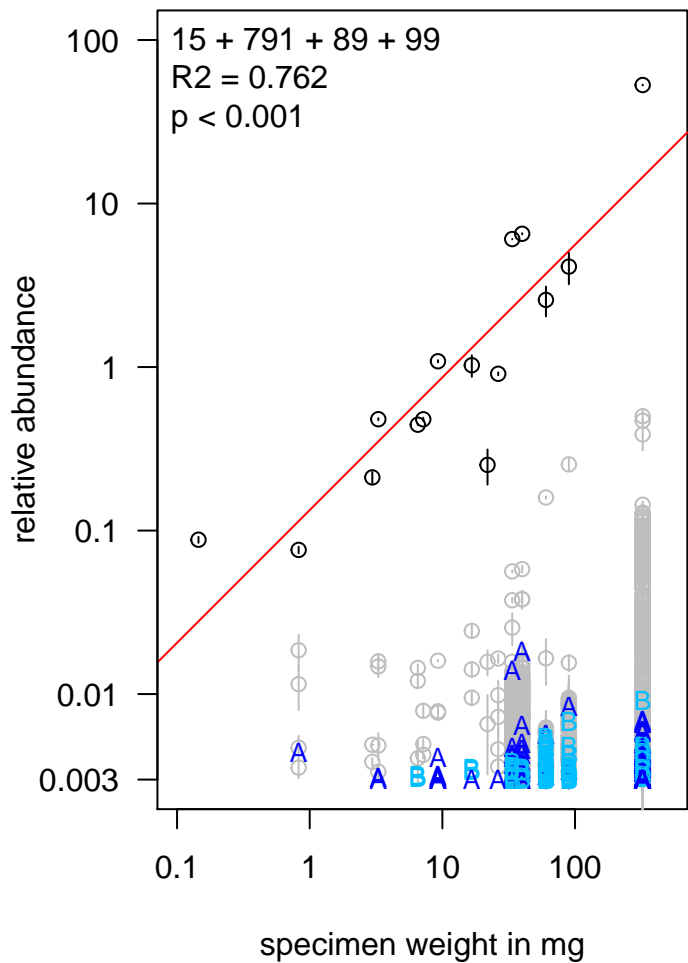

Supplement: Supplemental Information 8 [file peerj-06-4644-s008.zip › Scripts_S1_v3/1_Single_species_mock/R_dif_ee_filtering/1_tables/1_DceM__noDenoise.pdf]

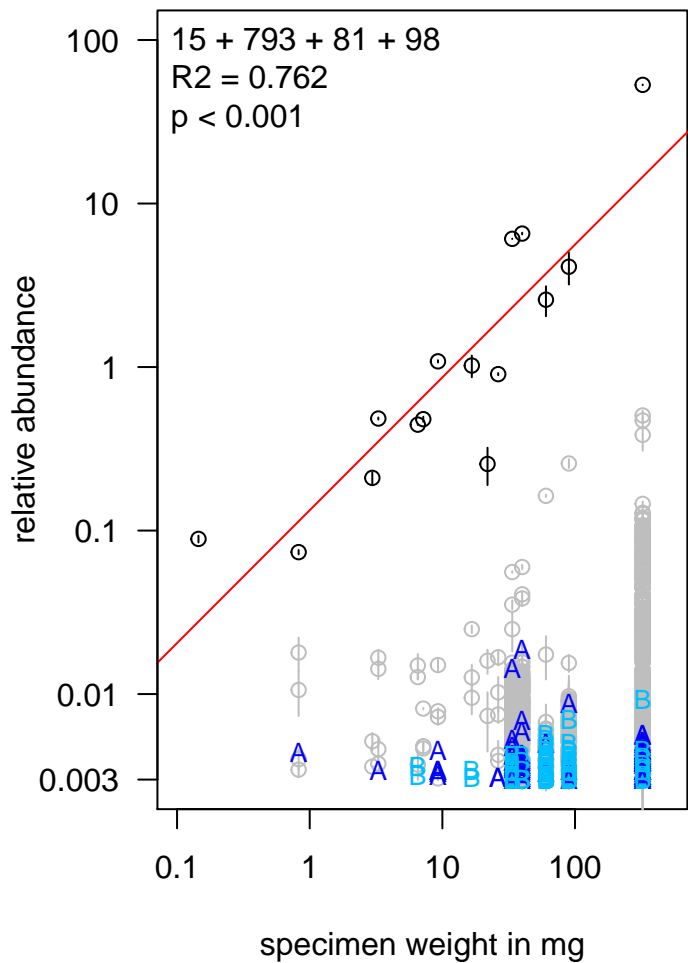

Supplement: Supplemental Information 8 [file peerj-06-4644-s008.zip › Scripts_S1_v3/1_Single_species_mock/R_dif_ee_filtering/1_tables/2_DceM__ee2_noDenoise.pdf]

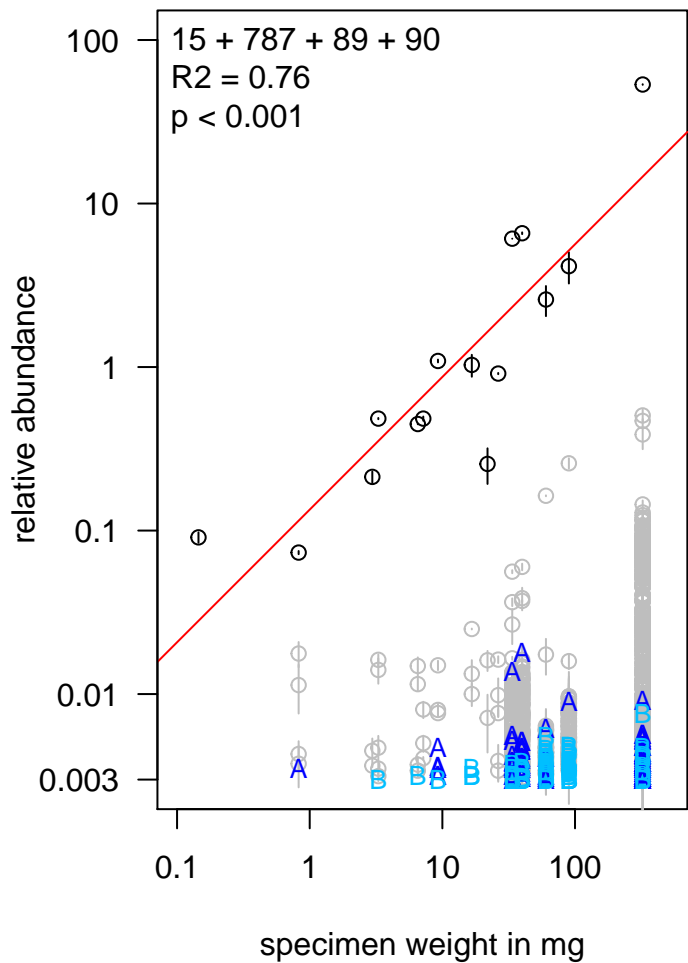

Supplement: Supplemental Information 8 [file peerj-06-4644-s008.zip › Scripts_S1_v3/1_Single_species_mock/R_dif_ee_filtering/1_tables/3_DceM__ee1_noDenoise.pdf]

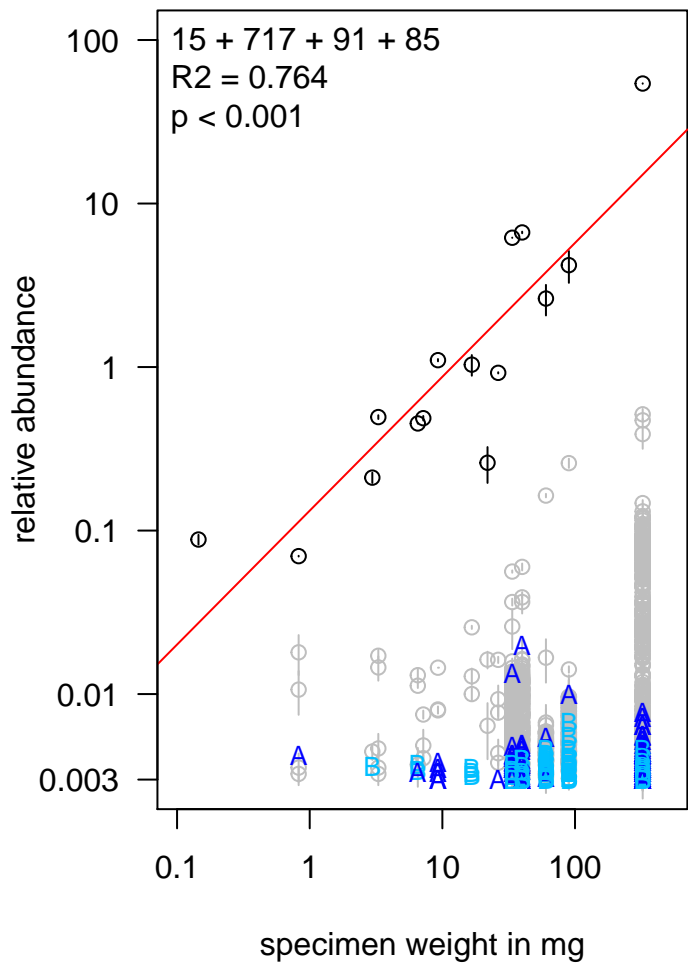

Supplement: Supplemental Information 8 [file peerj-06-4644-s008.zip › Scripts_S1_v3/1_Single_species_mock/R_dif_ee_filtering/1_tables/5_DceM__ee0.2_noDenoise.pdf]

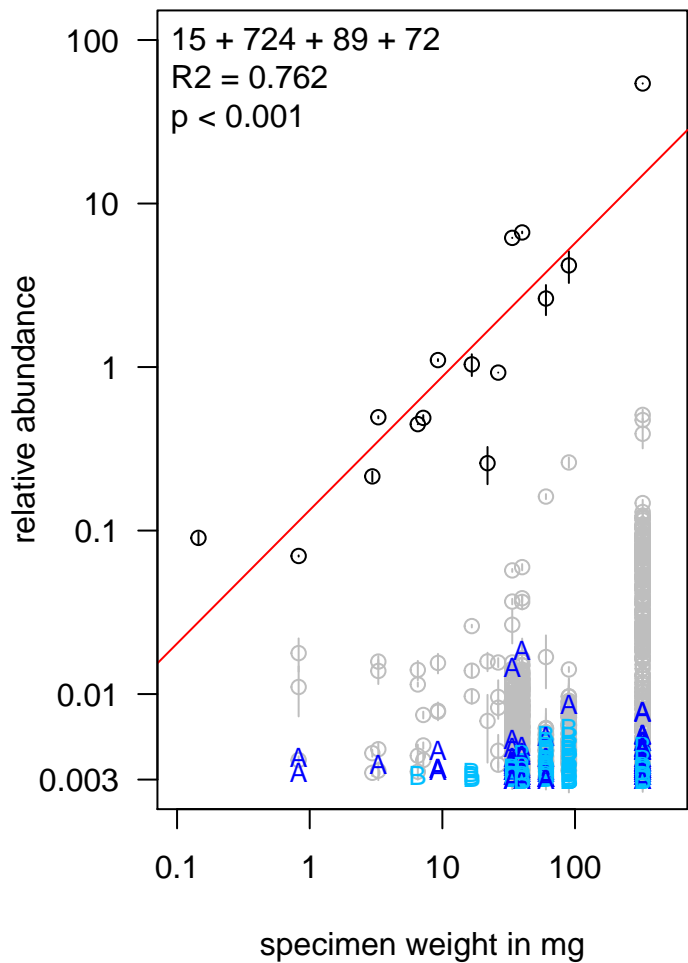

Supplement: Supplemental Information 8 [file peerj-06-4644-s008.zip › Scripts_S1_v3/1_Single_species_mock/R_dif_ee_filtering/1_tables/6_DceM__ee0.1_noDenoise.pdf]

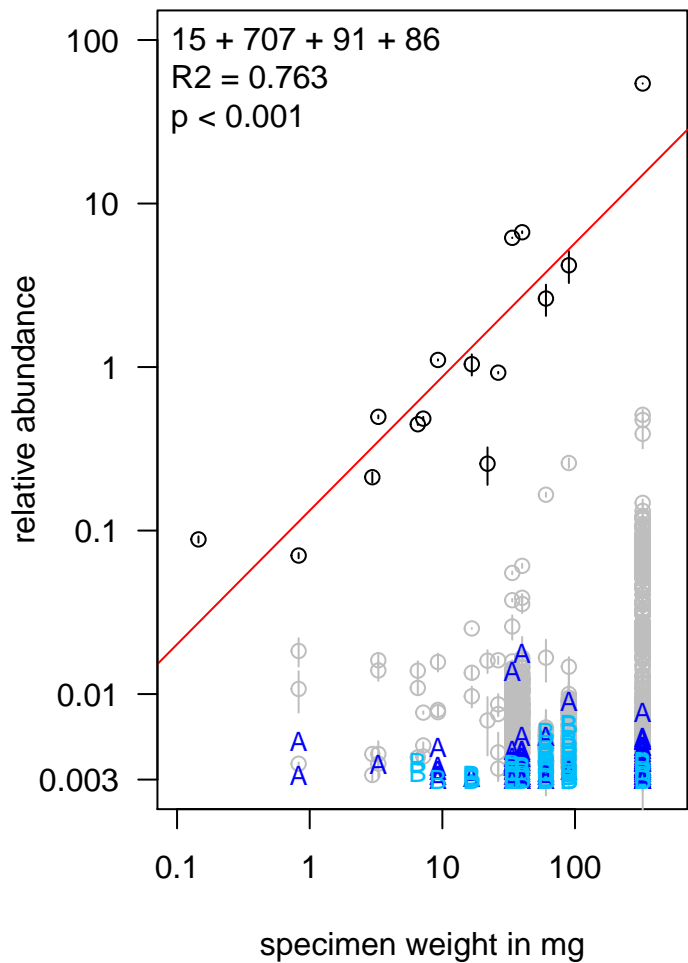

Supplement: Supplemental Information 8 [file peerj-06-4644-s008.zip › Scripts_S1_v3/1_Single_species_mock/R_dif_ee_filtering/1_tables/7_DceM__ee0.05_noDenoise.pdf]

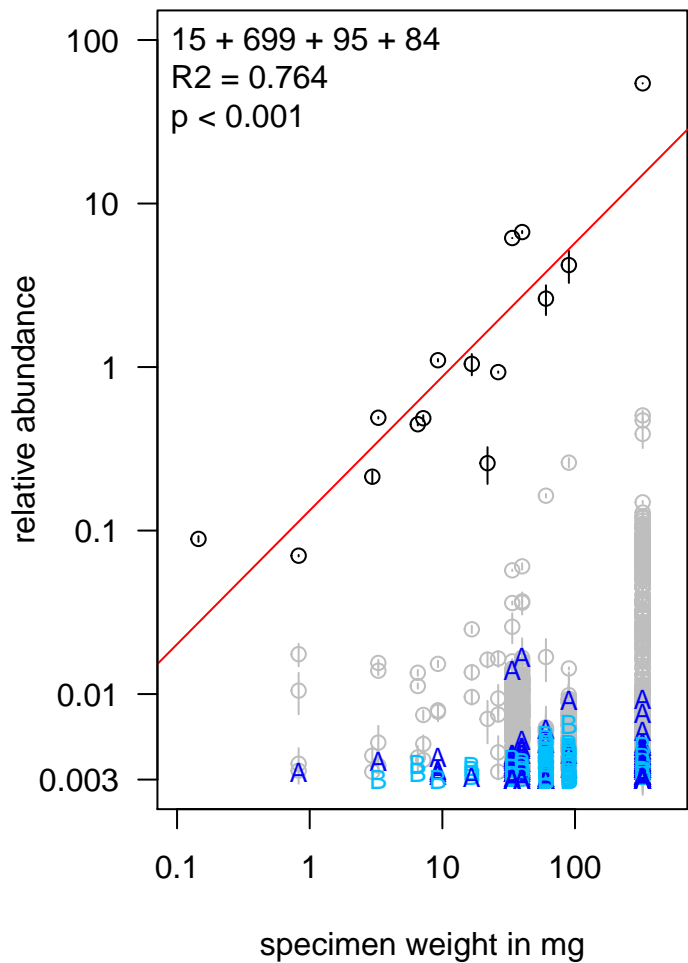

Supplement: Supplemental Information 8 [file peerj-06-4644-s008.zip › Scripts_S1_v3/1_Single_species_mock/R_dif_ee_filtering/1_tables/8_DceM__ee0.02_noDenoise.pdf]

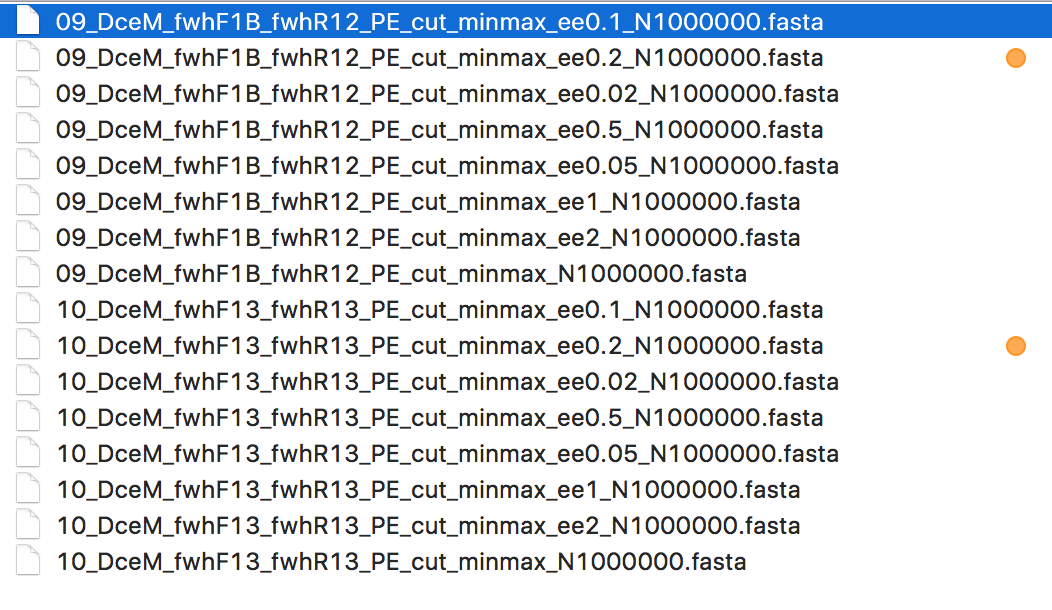

Supplement: Supplemental Information 8 [file peerj-06-4644-s008.zip › Scripts_S1_v3/1_Single_species_mock/R_dif_ee_filtering/_data/Screen Shot 2018-03-14 at 09.21.12.png]

color\_guide

|    |              |                                                                                   |
|----|--------------|-----------------------------------------------------------------------------------|
| 1  | #E41A1C      | 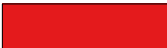 |
| 2  | #377EB8      | 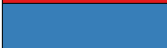 |
| 3  | #4DAF4A      | 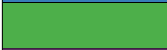 |
| 4  | #984EA3      | 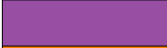 |
| 5  | #FF7F00      | 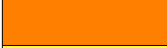 |
| 6  | #FFFF33      | 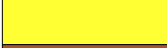 |
| 7  | #A65628      | 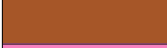 |
| 8  | #F781BF      | 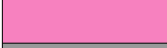 |
| 9  | #999999      | 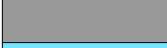 |
| 10 | lightskyblue | 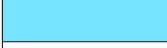 |
| 11 | white        | 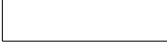 |

Supplement: Supplemental Information 8 [file peerj-06-4644-s008.zip › Scripts_S1_v3/2_Monitoring_samples/r maps/2 selected MAPS/color_guide.pdf]

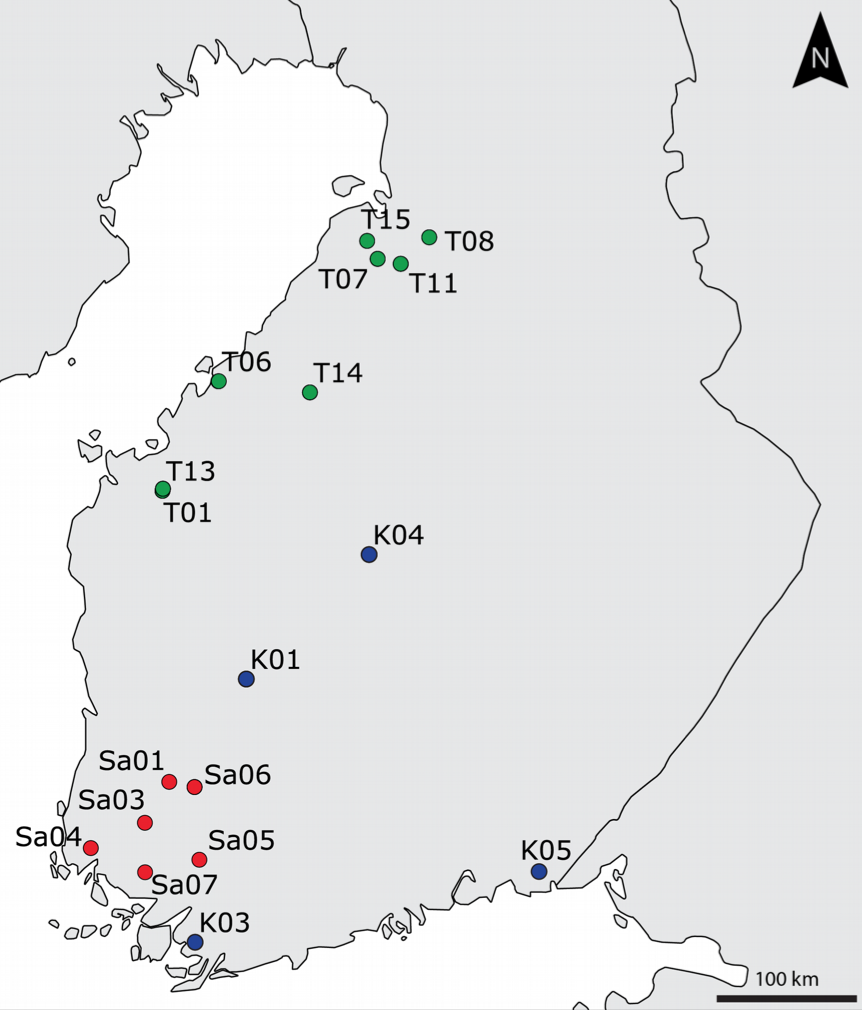

Supplement: Supplemental Information 8 [file peerj-06-4644-s008.zip › Scripts_S1_v3/2_Monitoring_samples/r maps/map_finland.png]
